# Insights into European Drug Shortages: A Survey of Hospital Pharmacists

**DOI:** 10.1371/journal.pone.0119322

**Published:** 2015-03-16

**Authors:** Kim Pauwels, Steven Simoens, Minne Casteels, Isabelle Huys

**Affiliations:** KU Leuven Department of Pharmaceutical and Pharmacological Sciences, 3000, Leuven, Belgium; University of Calgary, CANADA

## Abstract

Drug shortages are a complex and global phenomenon. When a drug cannot be delivered at the moment of patient demand, every stakeholder in the health care system is affected. The aim of this study was to investigate the characteristics, clinical impact, financial impact and management of drug shortages in European hospital pharmacies and identify opportunities for prevention and mitigation of drug shortages in Europe. An online survey was designed based on a review of the literature and interviews and was sent to subscribers of *Hospital Pharmacy Europe* between June and September 2013. Forty-five percent of respondents (n = 161) indicated that life sustaining or life preserving drugs such as oncology drugs were affected by drug shortages. More than 30% of respondents indicated that drug shortages in Europe were always or often associated with increased costs for hospitals, increased personnel costs and more expensive alternative drugs (n = 161). On the question when information about a drug shortage was obtained, 42% of respondents answered that information from the pharmaceutical company was obtained at the time of no delivery, 50% indicated that information from the wholesaler was obtained at the time of no delivery, while 40% of respondents indicated that information was never or rarely received from the government (n = 161). Fifty seven percent of respondents strongly agreed that an obligation to the producer to notify further shortages could help to solve the problem (n = 161). These results showed that pharmaceutical companies and wholesalers are already involved in the management of drug shortages, while a role is still reserved for the government. Mandatory notification in advance and centralized information can help to reduce workload for hospital pharmacists, will allow early anticipation of drug shortages and will facilitate mitigation of the clinical impact on patients.

## Introduction

A drug shortage can be defined as a shortcoming in the supply of a medicinal product that affects the patient’s ability to access the required treatment in due time. The origins of drug shortages are complex and diverse and the problem can be situated at both the supply and demand side [1,2]. Drug shortages affect every stakeholder of the health care system and collaborative efforts are required to manage and mitigate shortages [3]. Drug shortages are likely to affect workload and clinical decision-making and a clinical and financial impact needs to be anticipated [4,5].

An inquiry of United States (US) health care workers showed that in up to 99% of surveyed hospitals, drug shortages were experienced in the months preceding that study [6–8]. Pharmacists are often the first health care professionals who will be faced with the shortage. They will start seeking solutions for the problem and initiate negotiations with manufacturers and wholesalers. Furthermore, physicians and nurses need to be informed to consider future treatment options [3]. The drug in short supply can often be substituted by alternative drugs but in some cases such as for chemotherapy or antibiotics, alternatives are of little help [9]. Alternatives potentially have lower efficacy or suboptimal safety profiles and deliberation about alternative agents with physicians, nurses and other health care workers is required [10]. Delayed treatment or switches to alternative therapies can result in disease progression, increased risk for adverse effects or medication errors and reduced compliance [4,5]. Although all disease domains are affected by shortages, many of the drugs involved in US drug shortages are considered to be high alert medications such as heparin and propofol [3,6,11]. Their shortage does not only impact patient safety but also hospital performance in cases where treatment or surgery needs to be postponed, cancelled or even transferred to other hospitals where the necessary treatments are still available [6,8]. Surveys conducted in the US revealed that pharmacists often learn about the shortage at the moment they fail to receive an ordered product from the wholesaler or manufacturer (i.e. at the time of no delivery) [8]. Besides lack of advanced notification of the drug shortage, health care workers mentioned that the information they received about shortages was often inadequate [12]. Labor costs associated with managing drug shortages are estimated at $216 million annually in the U.S. [12]. The financial impact of drug shortages where generic equivalent alternatives are available exceeds $78 million with the highest cost impacts coming from infectious diseases, surgery, oncology and cardiovascular diseases [1]. The purchase of overall equivalent or alternative therapeutic substitutes is estimated to cost US hospitals about 215$ million annually [10].

Despite numerous articles appearing in the pharmaceutical trade magazines, facts and figures about drug shortages in Europe are rarely reported in the scientific literature [11–16]. Studies about the effect of drug shortages in European hospitals are limited. In 2012, the European Association of Hospital Pharmacists (EAHP) launched a questionnaire concerning drug shortages in European hospitals [17]. The questionnaire results revealed that 99% of hospital pharmacists experienced shortages in the past year [19]. According to the respondents, shortages occurred weekly and sometimes daily and the problem has emerged over the past years. On the question of whether patient care is affected by shortages, opinions are diverse with 50% of respondents saying it is affected while another 46% of respondents indicated that the hospital is able to manage the problem without the patient being affected [19]. Nevertheless, it cannot be neglected that drug shortages are also affecting European hospitals and that this puts the patients at risk. The hurdles in the management of drug shortages need to be identified and conquered to allow optimal patient care. Therefore the aim of this study was to further investigate the characteristics, impact, causes and management of drug shortages in European hospital pharmacies and identify opportunities for prevention and mitigation of drug shortages in Europe.

## Methods

Because of the nature of the survey, it was not required to seek approval from a research ethics committee. Data were analyzed anonymously.

A survey about drug shortages in European hospital pharmacies was developed based on a review of the literature [1,3,6,7,17–20] and five interviews with Belgian pharmacists. The online survey was setup in SurveyMonkey (2013) and tested by three authors of the manuscript, one student involved in the project, three affiliates of the project sponsor and the members of the editorial board of Hospital Pharmacy Europe (HPE) to collect comments about the length of the survey and clarity of the questions. The final survey included 29 questions. The survey enquired about the characteristics of the hospitals where respondents work, whether particular drug classes are more affected by shortages than others, which disease domains are affected by drug shortages and how shortages in particular disease domains can be characterized. Additionally, the clinical and financial impact, reported causes, measures to handle shortages, channels to obtain information about the drug shortage and possible solutions were questioned. The respondents were asked to estimate time spent per week (number of hours) on the management of drug shortages. A copy of the survey can be obtained upon request.

The survey was launched in the May-June issue of HPE which was distributed on the first of June 2013 to 12,000 subscribers. The first response was received on 7 June 2013. From then on, a link to the survey was announced in the weekly online bulletin of HPE, sent to 7,000 subscribers. The survey was closed on 15 September 2013.

The answers of respondents employed in European countries who completed the first four questions of the survey (country of origin, type of hospital (acute/university/psychiatric), size of hospital and the question “do you have the impression that particular drug types suffer more from shortages than others?”) were considered for further analysis.

Only descriptive statistics were performed. Characteristics of drug shortages were compared between different geographical regions i.e. Northern Europe, Eastern Europe, Southern Europe and Western Europe, based on the classification of European countries by the United Nations Statistic Division [21]. When at least ten respondents of the same country fulfilled the requirements for inclusion as described above, the country was additionally included in the analysis as such. Clinical and financial impact, causes for drug shortages and measures to handle drug shortages and possible solutions were analyzed for overall respondents. Relative numbers were presented as percentages and the considered sample size (n) was included in the result. The respondents were also asked to estimate the time spent on different items related to management of shortages. The number of hours per week spent on the management of drug shortages was estimated by the median number of hours obtained over all answers.

## Results

### Characteristics of respondents

Two hundred and eighty respondents participated in the survey. Of these, 161 respondents were retained for further analysis based on relevance of their country of origin for this study (e.g. European country) and completion of the first five questions of the survey. Twenty nine percent of European respondents were working in a hospital in Western Europe, 34% in Northern Europe, 21% in Southern Europe and 16% in Eastern Europe (n = 161). An analysis was also performed for Belgium (n = 11), the Netherlands (n = 16) and United Kingdom (UK) (n = 44). The majority of the European respondents reported working in an acute hospital (56%), while 37% were working in a university hospital and 7% were based in a psychiatric hospital (n = 161).

### Characteristics of drug shortages

**Fig 1:**
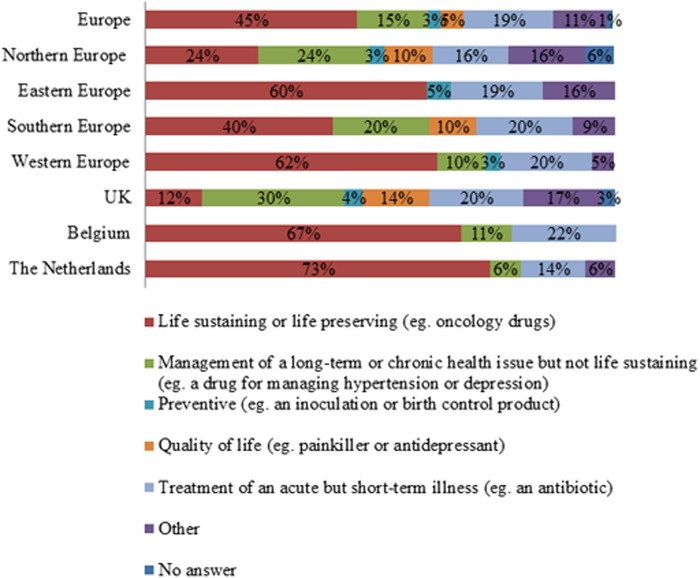
Drug types affected by drug shortages according to the respondents. Respondents who indicated that particular types of medicines suffered more from shortages than others were considered. The relative number of respondents per answer was shown for Europe (n = 128), Northern Europe (n = 8), Eastern Europe (n = 20), Southern Europe (n = 30), Western Europe (n = 16), the UK (n = 29), Belgium (n = 9) and the Netherlands (n = 15).

**Fig 2:**
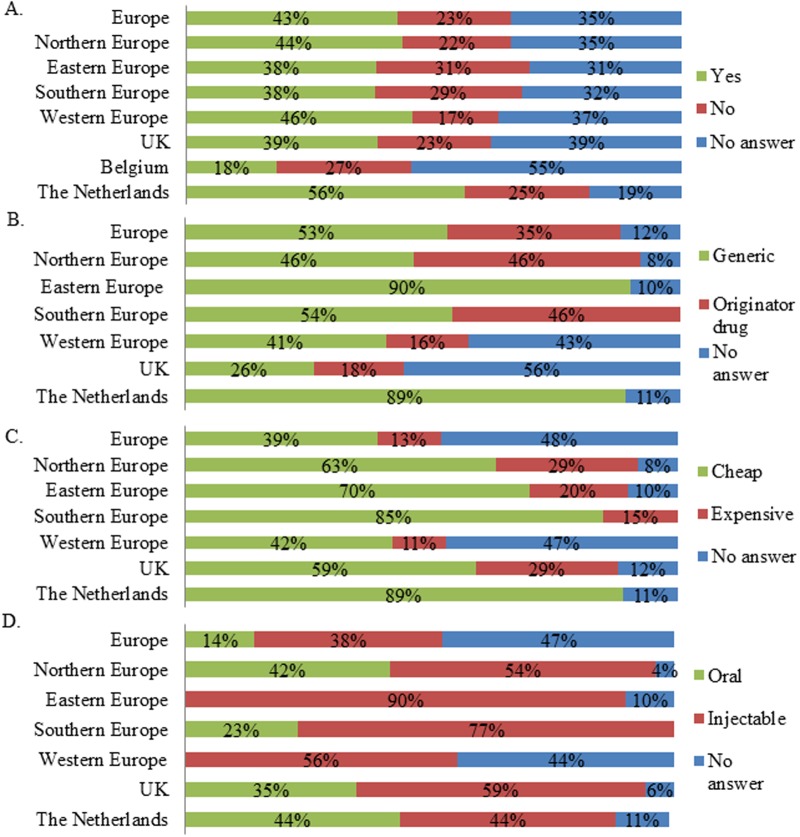
Characteristics of drug shortages for anti-infective drugs per region and country. A. Answers to the question “Are anti-infective drugs affected by drug shortages?”. The relative number of respondents per answer was shown for Europe (n = 161), Northern Europe (n = 55), Eastern Europe (n = 26), Southern Europe (n = 34), Western Europe (n = 46), the UK (n = 44), Belgium (n = 11) and the Netherlands (n = 16). B. Answers to the question “If yes, are these affected drugs mostly generic or originator drugs?”. C. Answers to the question “Are these affected drugs mostly cheap or expensive?”. D. Answers to the question “What is the most affected form?”. For Figs. B-D, respondents who indicated the disease domain is affected were considered. The relative number of respondents was shown for Europe (n = 69), Northern Europe (n = 24), Eastern Europe (n = 10), Southern Europe (n = 13), Western Europe (n = 21), the UK (n = 17), the Netherlands (n = 9).

**Fig 3:**
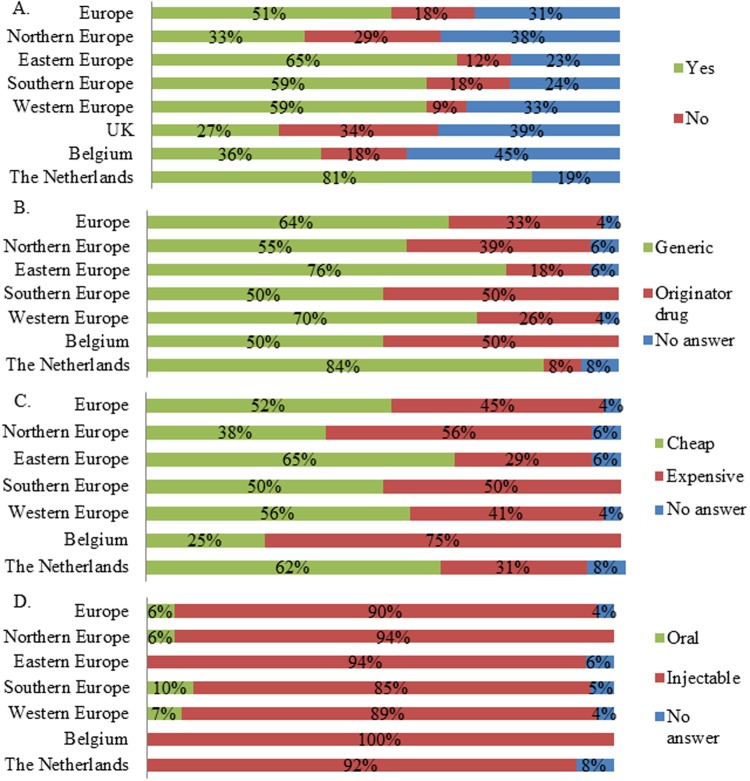
Characteristics of drug shortages for cancer drugs per region and country. A. Answers to the question “Are cancer drugs affected by drug shortages?”. The relative number of respondents per answer was shown for Europe (n = 161), Northern Europe (n = 55), Eastern Europe (n = 26), Southern Europe (n = 34), Western Europe (n = 46), the UK (n = 44), Belgium (n = 11) and the Netherlands (n = 16). B. Answers to the question “If yes, are these affected drugs mostly generic or originator drugs?”. C. Answers to the question “Are these affected drugs mostly cheap or expensive?”. D. Answers to the question “What is the most affected form?”. For Figs. B-D, only respondents who indicated the disease domain were affected by shortages were considered. The relative number of respondents was shown for Europe (n = 83), Northern Europe (n = 18), Eastern Europe (n = 17), Southern Europe (n = 20), Western Europe (n = 27), Belgium (n = 4), the Netherlands (n = 13).

Seventy nine percent of respondents indicated that particular drug classes were more affected by shortages than others (n = 161). In Europe, the largest share of respondents indicated life preserving and life sustaining drugs were most affected by drug shortages (Fig. 1). This was also reflected in the regions and countries, except for Northern Europe and the UK (Fig. 1). Here an important number of respondents indicated that drugs for management of long-term or chronic health issues suffered from shortages (Fig. 1). Next it was investigated in which disease domain drugs were affected by shortages. Anti-infective drugs and cancer drugs were indicated by the majority of respondents as being affected by drugs shortages in Europe (Figs. 2–3). The drug shortages were specified as generic, cheap drugs for injectable administration (Figs. 2–3). Similar results were obtained for most regions and countries even though a large part of the UK respondents indicated that cancer drugs were not affected by shortages (Fig. 3). Drugs for cardiovascular diseases were affected by shortages according to a large share of respondents from Northern Europe (33% indicated “yes”, 27% indicated “no” and 40% provided no answer, n = 55), UK (34% indicated “yes”, 25% indicated “no” and 41% provided no answer, n = 44) and the Netherlands (50% indicated “yes”, 44% indicated “no” and 6% provided no answer, n = 16). Drugs for the central nervous system were reported to suffer from shortages by a large share of respondents from Northern Europe (51% indicated “yes”, 27% indicated “no” and 22% provided no answer, n = 55), UK (55% indicated “yes”, 23% indicated “no” and 23% provided no answer, n = 44) and Eastern Europe (35% indicated “yes”, 27% indicated “no” and 38% provided no answer, n = 26). For immunological products and vaccines, shortages were indicated by a major share of respondents from Southern Europe (35% indicated “yes”, 29% indicated “no” and 35% provided no answer, n = 34), Western Europe (33% indicated “yes”, 26% indicated “no” and 41% provided no answer, n = 46) and the Netherlands (44% indicated “yes”, 31% indicated “no” and 25% provided no answer, n = 16).

### Impact of drug shortages

**Fig 4:**
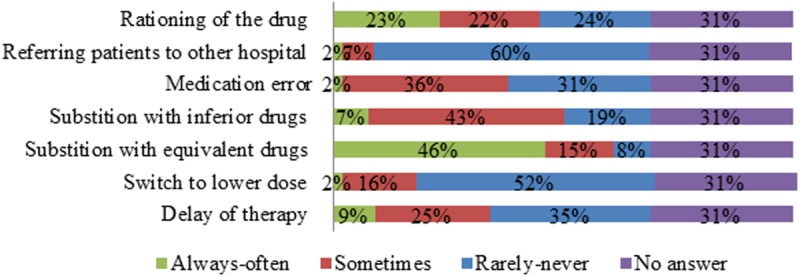
Answers to the question “What clinical impact has a drug shortage already caused in your hospital?” The relative number of respondents per answer was shown (n = 161). The answer “always” and “often” as well as “never” and “rarely” were grouped for the sake of clarity.

**Fig 5:**
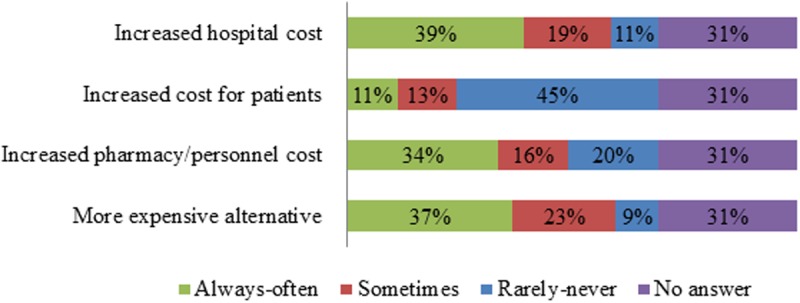
Answers to the question “Which financial consequence has a drug shortage already caused in your hospital?” The relative number of respondents per answer was shown (n = 161). The answer “always” and “often” as well as “never” and “rarely” were grouped for the sake of clarity.

According to Fig. 4, a majority of respondents indicated that patients were rarely or never referred to another hospital due to drug shortages. Further, most respondents indicated drug shortages rarely or never led to a switch to a lower dose (Fig. 4). Substitution with equivalent drugs was indicated to occur always or often by a large share of respondents, while medication errors and substitution with inferior drugs occurred sometimes according to a major share of respondents (Fig. 4). The opinions with regard to rationing of the drug and delay of therapy were more diverse (Fig. 4). On the financial level, increased hospital costs, increased pharmacy or personnel costs and the use of more expensive alternatives were indicated by a majority of respondents as consequences of drug shortages that occur always or often (Fig. 5). Cost for patients was indicated by a large share of respondents as being rarely or never affected by drug shortages (Fig. 5).

Thirty seven percent of respondents indicated that the personnel stress was (very) severely affected by shortages while 22% indicated that this was moderately affected, 10% of respondents indicated this was not/little affected and 37% of respondents provided no answer (n = 161).

The total time spent on the management of shortages by hospital pharmacists was estimated to be 12.8 hours/week. Most time was spent on tracking shortages (2.2 hours/week). Identifying therapeutic alternatives, purchasing therapeutic alternatives, deliberating with physicians and changing stock each required 2.0 hours/week. The development of policies required 1.6 hours/week while only one hour per week was spent on education of nursing staff. Of the 161 respondents, 35% of respondents provided no answer on this question.

### Causes of drug shortages

**Fig 6:**
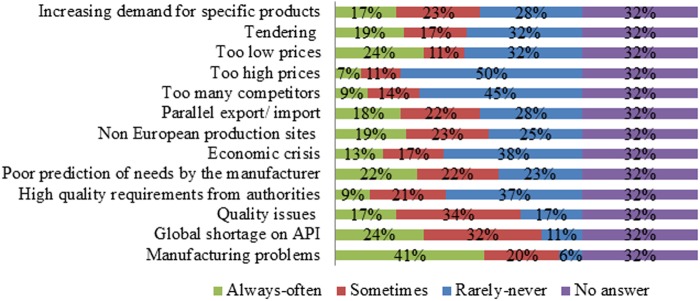
Answers to the question “What do you think are the causes for drug shortages over the last 12 months?” The relative number of respondents per answer was shown (n = 161). The answer “always” and “often” as well as “never” and “rarely” are grouped for the sake of clarity.

A majority of respondents believed that manufacturing problems were always or often the cause of drug shortages (Fig. 6). Too high prices, too many competitors, the economic crisis and high quality requirements from authorities were rarely or never the cause of drug shortages according to a large share of respondents (Fig. 6). For other possible causes, the answers were more diverse (Fig. 6). Tendering was estimated to play a role in drug shortages by the majority of respondents from Northern Europe (36% indicated “yes, 31% indicated “no” and 33% provided no answer, n = 55), Eastern Europe (27% indicated “yes”, 19% indicated “no” and 54% provided no answer, n = 26), the UK (36% indicated “yes”, 30% indicated “no” and 34% provided no answer, n = 44) and the Netherlands (50% indicated “yes”, 44% indicated “no” and 6% provided no answer, n = 16).

### Measures against drug shortages

A large share of respondents indicated that information was rarely or never received from a professional association (28% indicated “rarely-never”, 19% indicated “sometimes”, 16% indicated “often or always” and 37% provided no answer, n = 161), another hospital (28% indicated “rarely-never”, 22% indicated “sometimes”, 14% indicated “always or often” and 37% provided no answer, n = 161) or from the government (40% indicated “rarely-never”, 16% indicated “sometimes”, 7% indicated “always-often” and 37% provided no answer, n = 161). When asking respondents when they received information about a drug shortage from a professional association, most respondents indicated that they only obtained information at the moment they were willing to deliver the drug and they were not able to fulfill this task due to unavailability of the drug (28% indicated “at the time of no delivery”, 20% indicated “in advance”, 16% indicated “never” and 37% provided no answer, n = 161). Also other hospitals provided information at the moment hospital pharmacists faced a drug shortage (30% indicated “at the time of no delivery”, 16% indicated “in advance”, 18% indicated “never” and 37% provided no answer, n = 161). Information was often, always or sometimes obtained from a pharmaceutical company (26% indicated “always-often”, 25% indicated “sometimes”, 12% indicated “rarely-never” and 37% provided no answer, n = 161) and wholesalers (21% indicated “always-often”, 27% indicated “sometimes”, 16% indicated “rarely-never” and 37% provided no answer, n = 161), respondents indicated that also in this case, information was obtained at the time of no delivery for both pharmaceutical companies (42% indicated “at the time of no delivery”, 19% indicated “in advance”, 3% indicated “never” and 37% provided no answer, n = 161) as well as for wholesalers (50% indicated “at the time of no delivery”, 10% indicated “in advance”, 4% indicated “never” and 37% provided no answer, n = 161). Information provided by the government was never obtained according to a majority of respondents (40% indicated “rarely-never”, 16% indicated “sometimes”, 7% indicated “always-often” and 37% provided no answer). In order to obtain an equivalent product parallel import was always or often used by 16% to obtain an equivalent product and by 20% of respondents to obtain an alternative product, while a similar number of respondents indicated they use parallel import sometimes, rarely or never and 37% of the respondents provided no answer (n = 161).

A majority of respondents (54%) indicated an effort to solve the shortage was sometimes made by the manufacturer. Five percent indicated that the manufacturer never made an effort and four percent indicated that an effort to solve the shortage was always made by the manufacturer. Thirty seven percent of respondents did not answer this question (n = 161).

An obligation to the producer to notify further shortages could help to solve the problem according to more than half of the respondents (57% indicated “strongly agree”, 4% indicated “don’t know”, 2% indicated “(strongly) disagree” and 37% provided no answer, n = 161) while 52% of respondents (strongly) disagreed to reduce quality requirements (9% indicated “don’t know”, 2% indicated “(strongly) agree” and 37% provided no answer, n = 161).

## Discussion

Drug shortages are a complex and global problem [22]. While extensively studied in the US, drug shortages in European countries are yet little empirically investigated despite the fact that there are numerous reports that they frequently occur [11–15,23–25]. This study explores the phenomenon of drug shortages based on experiences of hospital pharmacists in a broad set of European countries. An inquiry into the characteristics of drug shortages showed that the typology of drug shortages differs between European regions and countries. Further, drug shortages in Europe were associated with a clinical and financial burden and additional workload for the hospital pharmacy. Perception of actions and measures already taken to manage shortages can reveal opportunities for further restriction of drug shortage impact on patients, healthcare workers and the hospital.

According to the results of this survey, drug shortages for life preserving and life sustaining drugs such as anti-infectives and cancer drugs are frequent in Europe. These findings are in accordance with a typology of US drug shortages presented by an IMS report [26]. Also other reports about drug shortages in US confirm shortages for anti-infectives such as antibiotics [3,6]. In a survey by the American Hospital Association (AHA), anesthetics and also drugs for emergency care are more often reported to be affected by drug shortages than anti-infectives and cancer drugs [7]. These shortages will affect health care workers as they can experience stress when providing surgery or emergency care, which can influence reporting of these shortages. In our study, drug shortages for anesthetics were only limitedly reported and the survey did not specifically question drug shortages in emergency care.

Shortages in Europe mostly involved cheap generic and injectable drugs. It is often assumed in the literature that injectable drugs are most susceptible to manufacturing problems or quality issues due to the complexity of the production process, high quality requirements and small margin for errors [2,20]. The susceptibility to drug shortages of generic injectables is sometimes linked to a lack of market attractiveness for generics [27,28]. A high price pressure would discourage marketing of generics and reduces investments in facilities and quality control, increasing susceptibility to what is perceived as manufacturing or quality issues, while economic issues might be the root of the problem [27,28]. The potential role of price and reimbursement policies on market attractiveness and availability of drugs in US is discussed in the literature [27,28]. The European market is shaped by regulations and policies at European and national level. Each country applies its own regulations for the supply and market access of pharmaceuticals, all having possible influences on the availability of drugs [29]. In European countries and regions, the typology of drug shortages is indeed heterogeneous. Respondents from the UK, the Netherlands and Eastern Europe indicated tendering as a cause of drug shortages. Tendering puts a high pressure on drug prices, especially when the number of competitors that participate in the tender is large. Furthermore, tendering can limit the number of suppliers, making drugs more susceptible to shortages when other suppliers need to start from zero to address a shortcoming in supply (e.g. capacity problem) [12,16,29,30]. According to the results of the survey, drug shortages in these tender markets were not limited to injectable drugs but also show up for the ‘less susceptible’ oral drugs such as drugs for cardiovascular diseases and the central nervous system. This strengthens the hypothesis that the cause of drug shortages goes beyond pure manufacturing problems related to technical issues or quality problems and can also involve economic factors. The European Directive 2004/18 from 31 March 2004 requires hospitals to purchase all supplies and services through public procurement [31]. On 15 January 2014, the criterion of most economically and advantageous tender (MEAT) was introduced to emphasize the importance of quality/price ratio of the offer instead of just accepting the lowest price. The new directive was expected to enter into force end of March 2014. In the Netherlands, the Royal Dutch Association for promotion of Pharmacy (KNMP) already developed an action plan to adjust the preferential policy (e.g. a tender-like system) and add a guarantee for sustainable supply to the conditions to win a tender [32].

Also parallel trade is blamed as a cause of drug shortages in Europe. The European market is a single market, which enables the free trade of goods, including pharmaceuticals, across the 28 Member States. After a patent holder has placed its products on the market, his intellectual property rights are exhausted and (cross border) trade with the product is allowed and can cause unforeseen export consequently leading to drug shortages [33]. Parallel trade has become important in the UK since the low value of the pound motivates wholesalers to buy products at a low price in the UK and sell them in high price countries such as Sweden and Germany [13,34]. Eastern Europe is even more characterized by the lowest prices among Europe, making countries in this jurisdiction an attractive market for export. In Poland, prices are on average 30% lower than in Germany [35]. Drug shortages for schizophrenia in Slovakia, where the drug price is set based on the second lowest price available in Europe, were also related to parallel trade [35–37]. Parallel trade is likely to particularly affect shortages of the patented medicines due to high prices of such medicines and bigger financial incentives for the wholesalers. In case of generics, large price disparities across the Member States can increase the role of parallel trade, particularly affecting countries with comparatively low prices of generic medicines [29]. Parallel trade was however poorly recognized by respondents in our study which can be explained by the fact that parallel trade affects the hospital drugs to a lesser extent than drugs distributed via community pharmacies. Hospital pharmacists presented manufacturing problems as the most important cause of drug shortages.

Although it was already indicated by previous studies that pharmacists are not systematically informed about the cause behind a shortage [8,17], manufacturing problems can still play a role irrespective of economic root causes presented above. For example, immunological products involve a large amount of biologically active ingredients which are unique and cannot be included in tendering. For classes such as immunological products, it is likely that real manufacturing issues or quality problems are involved in shortages [2,20].

Regardless of the cause, when a product cannot be delivered at the moment of patient demand, every stakeholder of the health care supply chain is affected. According to US studies, the purchase of alternative drugs causes additional costs for the hospital due to reimbursement restrictions [4,7]. Our study confirmed that the hospital itself is disadvantaged by drug shortages due to more expensive alternatives and additional hospital costs while patients remain free from financial burden. Patients will however not escape from a clinical impact. Substitution with alternative drugs can imply medication errors, adverse effects and disease progression. Drug shortages are related to clinical risks such as medication errors in this study as well as in other reports [7,8]. Other studies report a high number of “near miss errors”, including errors in dosing conversions and the preparation of wrong concentrations that never reach the patient [4,8]. Near miss errors were not included in this questionnaire and also patient outcomes were not surveyed. In addition, the pharmacy personnel is not always aware of clinical incidents and can underestimate the impact of drug shortages.

Pharmacists often succeed in obtaining an equivalent for the drug in shortage but this required a considerable amount of time. The workload associated with identification and purchase of alternative treatments might be reduced by increasing collaboration and sharing information between different hospitals, but the competitiveness between hospitals may make this unlikely. The median number of working hours to manage shortages estimated in this study is comparable to a survey carried out in the UK by the pharmacists’ organization *Chemist+Druggist* that previously showed that 95% of pharmacists spent between one and two hours per week on tracking out-of-stock drugs [14]. Another study conducted in US confirmed that pharmacists spent in total 9 hours per week to the management of drug shortages [3]. It was not always clear whether respondents considered the total number of working hours spent by their unit, or the number of hours per pharmacist. The workload related to drug shortages in general is underestimated because other health care workers such as physicians and nurses are also charged with the management of shortages [3]. Physicians are inevitably involved in deliberation about therapeutic alternatives while education of nursing staff was one of the actions to which the smallest amount of time was spent. According to other studies, nurses sense ignorance when they are not aware which alternative medications to consider and when they are unfamiliar with dosing and administration of alternative drugs in case of shortages [6,8]. Improving education of nursing staff might be one of the targets to limit the occurrence of medication errors.

Personnel stress suggests a high level of frustration and sense of responsibility by hospital pharmacists. Other studies relate a high level of frustration to the lack of notification in advance of drug shortages [8]. Although respondents indicated information was provided by pharmaceutical companies and wholesalers, information was not provided in advance. The government was limitedly involved in the management of a shortage. A mandatory early notification of drug shortages imposed by the government will allow health care workers to anticipate a drug shortage in an early stage. In this way, a financial burden on the hospital can be mitigated. Further, early notification will allow well-timed education of health care workers about the use of alternative drugs. As a result, clinical effects of drug shortages can be reduced. Further, the possible impact of cost-containment policies, such as tendering, on the availability of drugs needs to be considered and applied with respect to the sustainability of the market. Currently, insights into the causes of drug shortages are lacking and all players of the drug supply chain tent to blame each other and evade responsibility instead of taking action.

This study suffers from several limitations. Firstly, as this study assumes drug shortages can be influenced by national policies, the regions considered in this study can be too broad to detect country-dependent effects. Secondly, some terms used in the questionnaire are not further defined to respondents and might be subjected to different interpretations. This is particularly important with regard to causes of drug shortages as the term manufacturing problems may cover problems with economic root causes such as tendering. Thirdly, the response rate for particular questions was relatively low. This can be due to the length of the questionnaire as well as to the retrospective nature of particular questions which makes it difficult to answer.

## Conclusion

This study investigated the characteristics, impact, causes and management of drug shortages in European hospital pharmacies. A role for European and national policy measures related to the market access (e.g. tendering) and trade of pharmaceuticals (e.g. parallel trade) in the cause of drug shortages was suggested. Further, drug shortages in Europe are associated with a financial burden on hospitals, workload for the hospital pharmacy and clinical risks for patients. Based on our results, opportunities for better prevention and mitigation of drugs shortages in Europe could be identified. While pharmaceutical companies and wholesalers are already involved in providing information and solutions, a role is still left for the government. Mandatory early notification of drug shortages, imposed by the government can help to anticipate the clinical impact of shortages while centralization of information is likely to reduce the workload for hospital pharmacists.

## Supporting Information

S1 Dataset Shows the answers for each question per respondent (N = 161). When the respondent provided no answer on the question, this was indicated as “-“. “NR3 means that an answer on that particular question is not required/not relevant due to a negative answer on a former question.
(XLSX)
